# Tree Diversity Enhances Stand Carbon Storage but Not Leaf Area in a Subtropical Forest

**DOI:** 10.1371/journal.pone.0167771

**Published:** 2016-12-09

**Authors:** Nadia Castro-Izaguirre, Xiulian Chi, Martin Baruffol, Zhiyao Tang, Keping Ma, Bernhard Schmid, Pascal A. Niklaus

**Affiliations:** 1 Department of Evolutionary Biology and Environmental Studies, University of Zurich, Zurich, Switzerland; 2 Department of Ecology, College of Urban and Environmental Sciences, and Key Laboratory for Earth Surface Processes of Ministry of Education, Peking University, Beijing, China; 3 Institute of Botany, Chinese Academy of Sciences, Beijing, China; Chinese Academy of Forestry, CHINA

## Abstract

Research about biodiversity–productivity relationships has focused on herbaceous ecosystems, with results from tree field studies only recently beginning to emerge. Also, the latter are concentrated largely in the temperate zone. Tree species diversity generally is much higher in subtropical and tropical than in temperate or boreal forests, with reasons not fully understood. Niche overlap and thus complementarity in the use of resources that support productivity may be lower in forests than in herbaceous ecosystems, suggesting weaker productivity responses to diversity change in forests. We studied stand basal area, vertical structure, leaf area, and their relationship with tree species richness in a subtropical forest in south-east China. Permanent forest plots of 30 x 30 m were selected to span largely independent gradients in tree species richness and secondary successional age. Plots with higher tree species richness had a higher stand basal area. Also, stand basal area increases over a 4-year census interval were larger at high than at low diversity. These effects translated into increased carbon stocks in aboveground phytomass (estimated using allometric equations). A higher variability in tree height in more diverse plots suggested that these effects were facilitated by denser canopy packing due to architectural complementarity between species. In contrast, leaf area was not or even negatively affected by tree diversity, indicating a decoupling of carbon accumulation from leaf area. Alternatively, the same community leaf area might have assimilated more C per time interval in more than in less diverse plots because of differences in leaf turnover and productivity or because of differences in the display of leaves in vertical and horizontal space. Overall, our study suggests that in species-rich forests niche-based processes support a positive diversity–productivity relationship and that this translates into increased carbon storage in long-lived woody structures. Given the high growth rates of these forests during secondary succession, our results further indicate that a forest management promoting tree diversity after disturbance may accelerate CO_2_ sequestration from the atmosphere and thus be relevant in a climate-change context.

## Introduction

Forests are of paramount importance for global biogeochemical cycling and climate regulation. However, experimental studies about biodiversity–productivity relationships has mostly focused on herbaceous plant communities to date [1], with results from tree communities only beginning to emerge [2,3]. An alternative to manipulating tree species diversity experimentally is to study the correlation of diversity and productivity along natural gradients [4,5]. This approach is complementary to experimental studies and has the advantage that effects are assessed under more natural conditions in complex ecosystems with tree communities presenting a diverse demographic structure. Gamfeldt et al. [6] studied multiple ecosystem services including productivity in Scandinavian forest inventory plots and observed a positive correlation with tree species richness. Paquette and Messier [4] analyzed productivity in permanent forest plots spanning a large latitudinal gradient encompassing forest ranging from mixed temperate to boreal. Growth was positively related to species richness, even when correcting for effects of stand basal area (which in part is the result of growth, i.e. which could also have been driven by species richness). Vilà et al. [5] carried out a similar analysis including inventory data from eleven different European forest types and found that mixed stands exceeded the productivity of monospecific stands on average by 24%, with the exception of a single forest type (acidophilous oak) where no positive effect was found. Studying temperate old-growth forest plots, other authors [7,8] found decreases in productivity with species richness, which were driven by very strong species-identity effects in one study [8]. Generally, these studies illustrate the need to carefully control for effects of drivers of productivity and diversity that may vary among study plots, in particular if strong environmental gradients are included as is typically the case when large geographical ranges are covered. Also, survey type studies often leave the question unresolved about whether productivity drives diversity, e.g. through competitive interactions, or vice versa [9]. This ambiguity can be resolved to some extent by comparative studies in which field plots spanning gradients in species richness but otherwise uniform site conditions are selected [10].

Subtropical and tropical forests are generally more species rich than forests at higher latitude. The diversity of tree species coexisting in forest has been related to niche-based and to neutral processes. Neutral theory proposes that equalizing tradeoffs lead to the coexistence of species with similar niches and equal fitness in an integrated functional sense [11]. While debated, empirical evidence for the partitioning of resources such as light and nutrients among forest tree species indeed is limited [12,13]. Also, niche complementarity may often be higher among conspecific individuals than among populations of different species [14,15]. On the other hand, a study by Johnson et al. [16] suggests that negative density-dependent mechanisms are more important in species-rich, low-latitude forest, which might be related to a greater importance of biotic interactions in these systems. In any case, if high species richness in subtropical and tropical forests is not related to high interspecific niche complementarity, then no pronounced dependence of productivity on species richness should be expected in these forest ecosystems. However, most studies to date were carried out in temperate to boreal forest with comparatively low species richness [4,5,7,8,14,17], and thus little is known about the role of biodiversity for ecosystem functioning in tropical and subtropical forests.

Trees (and forest communities) are characterized by a pronounced functional separation of a long-lived woody biomass fraction that stores large amounts of carbon and a much smaller, more rapidly turning over photosynthetically active leaf fraction. As a consequence, carbon storage is decoupled from leaf area and photosynthesis to an extent not found in non-woody aboveground vegetation, possibly giving rise to distinct biodiversity–ecosystem functioning relationships. Most forest biodiversity studies to date have focused on stem growth as the standard metric recorded in forest inventory plots [4,5], or used stem size to determine aboveground biomass, estimated using allometric relations (e.g. [18]). Thus, there is little direct evidence of biodiversity effects on leaf area, or more generally on allocation of carbon to different organs. Despite their low biomass fraction, leaves are functionally important for ecosystem functions because they intercept light and thus are responsible for carbon assimilation and affect competition for light and gap dynamics [19]. Leaf area and its spatial distribution also are relevant for microclimate and rain interception, thus affecting the forest’s energy and water balance and also splash erosion [20]. Leaves further are central in nutrient storage and cycling and offer space and nutrition to herbivores and pathogens. In summary, it is conceivable that leaf area and leaf biomass show a different response to species richness than total and woody biomass do.

One potential mechanism driving biodiversity–productivity relationships is resource niche complementarity. However, plants thrive on a similar set of resources and it remains open to which degree there is the potential for interspecific resource partitioning. While light is important and shapes plant competitive interactions, stand-level productivity in forests is often limited by other factors including temperature, nutrients, and water [21]. Competition for light is strongly asymmetric, with taller individuals being able to pre-empt this resource, which limits the potential for vertical niche partitioning. Nevertheless, there is the possibility that more species-rich communities exhibit a higher diversity with respect to shade-tolerance, tree height, and canopy architecture [22], and that this facilitates, directly or indirectly, higher productivity.

In summary, little is known on biodiversity–productivity relationships in species-rich forest outside the temperate and boreal zone, and most forest studies have focused on stem growth-related metrics. Here, we present an analysis of carbon storage, leaf area, and vertical canopy structure in comparative study plots that were established in a highly diverse mixed broad-leaved forest in subtropical China [10,23]. These plots were deliberately selected to span a gradient in species richness in an otherwise relatively uniform nature reserve. In addition, plots also spanned a factorial gradient in secondary succession due to former timber harvesting by local farmers. We recorded leaf area index two times in a year by digital hemispheric photography, at the start and the peak of the growing season. We further tracked cohorts of trees on a per-individual basis over a total of four years and estimated the amounts of C bound in aboveground biomass. This data extend previous time series [10], and, more importantly, translate basal-area data onto a scale relevant in a biogeochemistry and climate-change context. Finally, we quantified the variation in tree size as metric of vertical canopy space use. Specifically, we were interested in testing (1) whether responses of species-rich subtropical forest to biodiversity were similar in magnitude to the ones reported for more species-poor temperate and boreal systems; (2) whether leaf area responses paralleled stem basal area responses, (3) whether more species-rich communities displayed a higher level of vertical canopy complexity, and whether this correlated to biomass and productivity.

## Material and Methods

### Study site and experimental design

We studied the effects of tree species richness and stand age on productivity and canopy structure over a 4-year period in Gutianshan National Nature Forest Reserve (GNNR), Zhejiang province, south-east China (29°15’N, 118°07’E). Permission to carry out this study was granted by the administration GNNR; the study did not affect endangered or protected species. The climate at the site is subtropical monsoon, with a mean annual air temperature of 15°C and a mean annual precipitation around 2000 mm. The vegetation is mixed deciduous to evergreen broad-leaved forest. A total of 1462 seed plant species belonging to 684 genera and 149 families are found in the 81 km^2^ reserve, with 258 woody species [24]. The number of evergreen and deciduous tree species is similar, but the former dominate by number of individuals. The reserve covers a mosaic of forest patches in different successional stages with five to more than 80 years since the last logging event. In 2008, 27 plots were established to cover separate gradients of tree species richness and stand age [23]. Plot were 30 x 30 m in size and distributed throughout the reserve with an average distance of about 3 km. Tree diversity was quantified as number of species found in the tree cohort with a stem diameter at breast height (DBH) of at least 10 cm. Stand age was determined based on the age of the fifth-largest tree of each plot (determined from a stem core) which then was binned into five age classes (20–40, 40–60, 60–80, 80–100, 100–120 years old with 4–7 plots per age class). This classification is more systematic than the one we had previously adopted and which contained some subjective elements; data are similar, however [10,23]. Two plots were lost in the course of the study due to illegal logging, so that the present study encompasses 25 plots only.

### Tree inventory

In summer 2008 and 2012, we censored all trees with a diameter of at least 10 cm. The diameter was measured at breast height (DBH) using a diameter tape or calipers. In 2012, we also measured tree height (parallel to the stem if the tree was leaning) of the trees with a hypsometer (Vertex III, Haglöf AB, Sweden). Stand basal area (SBA) was calculated as the sum of the basal area of the individual trees (m^2^ ha^-1^). Stand basal area increment (ΔSBA) was calculated as the increase in stand basal area of all trees recorded in the first census and the second census, i.e. excluding ingrowth into and disappearance due to mortality from the censored size cohort because the inclusion of such trees would have greatly increase stochastic variation in the measurements. We further calculated the standard deviation of tree height, which can serve as a proxy of vertical space use and layering of foliage [25].

### Stand leaf area

Leaf area index (LAI) was assessed in early spring and again in summer 2012, with evergreen leaves present on both dates but deciduous leaves only on the second. Plots were divided into nine equally-sized quadrats and a hemispherical photograph of the canopy taken in the center of each quadrat, at 1.5 m above ground (Nikon D3000 SLR camera, Sigma 4.5mm f/2.8 circular fish eye lens). The camera was mounted vertically upwards on a tripod with the top of the photographs oriented to the geographic north. Photos were acquired under overcast sky or before sunrise or after sunset when illumination was even. Aperture was set to f/5.6 and ISO sensitivity to 200. Four photographs were taken, the first at the automatic exposure read by the camera, and the three others underexposed by 1, 2 or 3 f-stops by varying shutter speed. For each point, we selected the picture with the highest exposure but without noticeable blooming effect. Photographs were then preprocessed to discriminate between vegetation and open sky, using a custom software that allowed to adjust for uneven sky illumination by defining light intensity thresholds for selected polygons that then were extrapolated based on Delauney triangulation (software by the last author, unpublished). Correct discrimination was carefully checked visually in all images. Leaf area index was estimated from these pre-processed images using Hemisfer 2.1 [26], using a weighted ellipsoidal method, a non-linearity correction for slope, and a total observed zenith angle of 75° divided into five conical sections spanning 15° of azimuth angle. Plot-level LAI was calculated as the average of LAI estimated from the nine quadrats, separately for spring and summer.

### Stand aboveground carbon stocks

Tree aboveground biomass (stem, branches, and leaves) were estimated using allometric equations developed for the study area or close geographic locations [27]. The equations relate diameter at breast height and/or height to aboveground biomass. If a tree had multiple stems, we calculated the biomass for each stem separately. Biomass was converted to carbon stock assuming a C content of 50%.

### Functional and phylogenetic diversity

Functional (FD sensu [28] and phylogenetic diversity (PD) were based on the functional trait dendrogram and phylogenetic tree from [10]. FD was based on the eight traits leaf seasonality (evergreen vs. deciduous), leaf habit (broadleaved vs. coniferous), specific leaf area (SLA), leaf carbon to nitrogen ratio, leaf size (dry weight of a typical mature leaf), the typical maximum height reached by mature individuals and stem-wood density. These traits were chosen because they are indicators of the allocation strategy and growth characteristics of tree species [29]. PD was based on phylogeny including 440 seed plant species present at our study site. Both FD and PD were calculated as total branch length in the sub-tree defined by the species occurring in a plot. We refer to the Supporting Information in [10] for method details.

### Statistical analysis

We tested for effects of tree species richness and stand age by fitting multiple regression models with sequential sum of squares (lm function of R 3.2; http://r-project.org). Because richness and stand age were correlated, we tested effects of species richness before and after adjusting for age (richness fitted before or after age). We then used structural equation models to explored the relationship among tree diversity (latent variable defined by species richness, functional diversity and phylogenetic diversity), stand age, and their indirect effects mediated by changes in tree density (i.e. the number of tree individuals found in a plot). These models were fitted by generalized least squares using lavaan (http://lavaan.ugent.be). The analyzed data is available as S1 Data.

## Results

In 2012 mean stand basal area was 24.83 m^2^ ha^-1^ (range 2.20–47.91), mean LAI_spring_ 2.83 (range 1.26–3.5), mean LAI_summer_ 3.3 (range 2.24–4.5), and mean C stock 63.42 Mg ha^-1^ (range 4.48–134.7). The number of trees with DBH ≥ 10 cm that were alive in 2008 and 2012 in a plot was on average 60 (range 10–96); the mean number of species was 11 (range 3–19). This corresponds to tree densities of 666.7 (range 111.1–1066. 7) trees per hectare.

### Stand basal area

**Fig 1:**
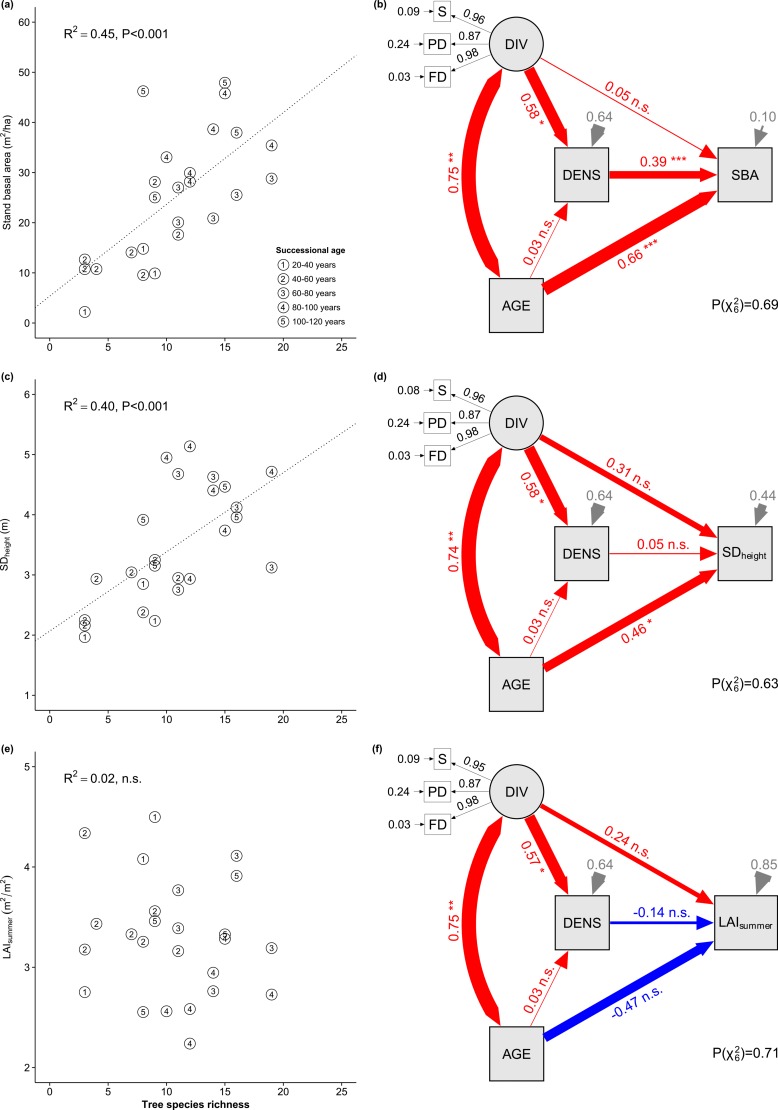
Stand basal area, vertical structure and leaf area as a function of tree species richness, stand age and tree density. Stand basal area (a), vertical structure, quantified as standard deviation of tree height (c), and leaf area index (e) as a function of tree species richness and stand age. Lines represent the linear regression between richness and the response variable (ignoring stand age). Structural equation models (SEM) for stand basal area (b), vertical structure (d) and leaf area (f) in dependence of stand age, tree diversity and tree stem density. Species richness enhanced wood production (SBA) and vertical structure but did not affect stand leaf area. Path diagrams indicate effects of tree species diversity on SBA, either directly or indirectly via increases in tree density. The diagrams show standardized path coefficients (red: positive; blue: negative) and associated statistical significances (*** P<0.001; ** P<0.01; *P<0.05; (*) P<0.1). Grey arrows indicate error terms of response variables. Thin black arrows indicate the loadings of indicator variables and their errors. Variable abbreviations: S = species richness, PD = phylogenetic diversity, FD = functional diversity, DIV = diversity (latent variable related to previous three), AGE = stand age, DENS = tree density, SBA = total stem basal area, SD_height_ = height variation, LAI_summer_ = LAI in summer. The goodness of fit of each model was assessed with chi-square tests. A P-value >0.05 indicate a good fit between the SEM model and the observed data (i.e. the observed and expected covariance matrices are not different).

Stand basal area (SBA; range 2.2–47.9 m^2^ ha^-1^) and its 4-year increment (ΔSBA; range 0.66–2.97 m^2^ ha^-1^) increased linearly with tree species richness (SBA: F_1,22_ = 49.2, P<0.001; ΔSBA: F_1,22_ = 9.9, P<0.01; multiple regression with sequential sum of squares and species richness fitted before stand age; Fig 1, S1 Fig). Although stand age and species richness were partly confounded, the effect of richness remained statistically significant when tested after adjusting for stand age (SBA: F_1,22_ = 5.6, P<0.05; ΔSBA: F_1,22_ = 7.8, P<0.05).

SBA increased with stand age (F_1,22_ = 80.5, P<0.001 and F_1,22_ = 37.0, P<0.001 when stand age was fitted before and after species richness, respectively; Fig 1). In contrast, ΔSBA did not or only marginally significantly depend on stand age (F_1,22_ = 2.27, P = 0.14 and F_1,22_ = 0.10, P = 0.75 for stand age fitted before and after species richness, respectively).

Structural equation models revealed that the effect of tree species richness on stand basal area was mediated by an increase in tree density, i.e. by the number of tree individuals present in the plot (path DIV—>DENS—>SBA in Fig 1), whereas the effect of diversity on ΔSBA was due to enhanced individual growth rather than increased tree numbers (S1 Fig). The structural equation models further indicated that the positive overall effect of stand age on SBA was driven by an increase in individual size rather than by an increase in the number of trees (path AGE—>SBA in Fig 1). ΔSBA was not affected by stand age, neither through a change in tree density (path AGE—>DENS—>ΔSBA; S1 Fig) nor through a change in individual growth (path AGE—>ΔSBA; S1 Fig).

### Stand vertical structure

SD_height_ increased with species richness (F_1,22_ = 19.6, P<0.001 for richness fitted before stand age; F_1,22_ = 4.7, P<0.05 for richness fitted after age; Fig 1) and with stand age (F_1,22_ = 21.7, P<0.001 for age fitted before richness; F_1,22_ = 6.9, P<0.05 for age fitted after richness). The corresponding structural equation model suggested the spread in tree height was independent of tree density but instead a direct consequence of larger species numbers and age (Fig 1). The plot-level variance in tree height originated from approximately similar contributions of intra- and interspecific contributions (variance components σ^2^_inter_ = 7.13 and σ^2^_intra_ = 9.67 m^2^). However, species richness effects on SD_height_ were driven essentially by interspecific variation (F_1,24_ = 4.7 and 6.2; P = 0.04 and 0.02; richness fitted before and after age), whereas no such effect was found for intraspecific variation (species with one individual per plot excluded from analysis).

### Leaf area index

Species richness did not affect LAI, regardless of whether it was measured in spring or summer and whether richness was tested before or after adjusting for stand age (Fig 1). LAI_summer_ decreased marginally significantly with stand age (F_1,22_ = 3.97, P = 0.06 and F_1,22_ = 4.21, P = 0.05 for stand age fitted before and after richness, respectively; Fig 1). Test findings were corroborated by the structural equation models.

### Tree aboveground carbon stocks

**Fig 2:**
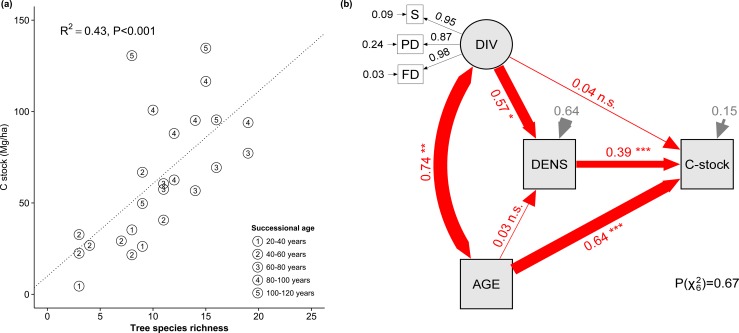
Carbon stocks in aboveground biomass. (a) Aboveground carbon stocks increased with tree species richness. (b) Structural equation model (SEM) linking C stocks to tree species diversity, stand age, and tree density. Variable abbreviations: S = species richness, PD = phylogenetic diversity, FD = functional diversity, DIV = diversity (latent variable related to previous three), AGE = stand age, DENS = tree density, C stock = C stored in the aboveground biomass. See legend of Fig 1 for details.

Carbon stock in aboveground biomass increased with tree species richness (F_1,22_ = 38.3, P<0.001 for richness fitted before stand age; F_1,22_ = 4.54, P<0.05 for richness fitted after stand age; Fig 2). Structural equation modelling indicated that this effect was mediated by an increase in tree density (path DIV—>DENS—>C, Fig 2). Carbon stock also increased with stand age but this effect was mediated primarily by increased individual size (path AGE—>C stock) rather than by an increased number of trees.

## Discussion

Our analyses of biodiversity–productivity relationships in species-rich subtropical forest indicated that more diverse stands had higher stand basal area and associated aboveground C stocks. Effects on stand basal area were paralleled by increased growth rates, i.e. stand basal area increases throughout the observation period correlated positively with tree species richness. The plot-level increases in biomass and productivity were accompanied by more variable tree height, i.e. better vertical canopy space use. Interestingly, leaf area did not change with species richness, suggesting a decoupling of the responses of wood and leaf production.

The observed effects of tree species richness on aboveground biomass increments are in line with previous observations [10], indicating that these effects are robust and persist over time. Our forest stands stored 4.5–135 Mg C ha^-1^ in aboveground biomass, which is in the typical range for subtropical evergreen broad-leaved forests of China [27]. Carbon stocks increased by 50 Mg C ha^-1^ per 10 extra species, or by +83% when doubling species number from 10 to 20. These effect sizes (Zr of 0.44–1.1 depending whether species richness is fitted before or after stand age) are larger than most species richness effects found in grassland biodiversity experiments [30] and forest plantations [31], and in agreement with recent studies in highly diverse tropical forests [32,33]. Given that these figures exceed the phytomass of herbaceous ecosystems by several orders of magnitude, such effects appear critical for vegetation–climate feedback under biodiversity change. In herbaceous ecosystems, most carbon is found in soil organic matter [34,35]; sustained long-term transfer of plant productivity to this pool could thus nevertheless lead to the accumulation of large amounts of C in the ecosystem despite limited storage in phytomass. Similarly, forests store large amounts of carbon belowground. In our study, no root biomass data was available; however, ecosystem-level C stock responses likely followed the patterns found aboveground, for several reasons. First, aboveground biomass generally exceeds belowground biomass in forests; for the type of forest we investigated a root-to-shoot biomass ratio of ca. ¼ is quite typical [36,37]. Second, allometric considerations suggest that a higher stand basal area also corresponds to larger belowground biomass [38,39], i.e. the C sequestration in aboveground biomass we report may in fact be underestimates of whole plant responses. Third, increased primary productivity can translate into larger carbon storage in soil organic matter, which would further exacerbate these carbon sequestration rates. Substantial C sequestration rates have been documented for the type of forest we investigated [40,41]. Our results thus suggest that a management of forest that endorses the conservation of biodiversity could accelerate CO_2_ removal from the atmosphere, at least after disturbance. In China, forest area and C sequestration have increased by 0.51% and 0.85% annually over the past three decades, mainly due to national reforestation and afforestation programs, but these focused on monoculture or low diversity plantations and further stimulations of C sequestration might be possible [41].

The strong, positive relation of tree species richness with woody biomass and growth suggests that niche-driven mechanisms were at play. The linear character of the relationship further indicates that functional redundancy did not substantially increase along the diversity gradient present in our study [42,43], which contrasts asymptotic, saturating responses of carbon storage in grassland and other forests [17,44,45]. The nature of complementarity that drives these patterns, however, remains unclear. Interspecific (but not intraspecific) variance in tree height increased in more species-rich plots, indicating that higher productivity was related to vertically more complexly structured canopies. This promotes the idea that more species-rich stands allow for a denser packing of woody biomass, i.e. that architectural complementarity is important in efficiently using canopy space for aboveground carbon storage. In European forest inventory plots, more species rich tree stands also were found to have canopies with more densely packed crowns [14]; unlike in our study, vertical stratification remained unaffected, but more species-rich plots did not have a higher stand basal area.

Interestingly, leaf area index did not respond to species richness, i.e. it did not reveal the effects of diversity that we observed for stand basal area or basal area growth. This might indicate that more diverse stands were able to intercept more light at a given LAI, or use the intercepted light more efficiently. Such effects might result from a reduced clumping of leaf area, or from increased photosynthetic light use efficiency [46,47]. However, we think that the more likely explanation is that allocation patterns and turnover rates of woody biomass are more important determinants of stand-level carbon accumulation than leaf area (and photosynthesis) per se. While light is important in shaping competition of neighboring trees and gap dynamics, system-level forest productivity often is limited by other resources including water and nutrients [21]. Photosynthesis then merely tracks growth which ultimately is controlled by other processes [48,49,50,51]. Under such conditions, partitioning of resources other than light may be more important in shaping biodiversity–productivity relationships. Light also is largely a directional resource that can be pre-empted by leaves at the top of the canopy. This asymmetry in competition likely reduces the possibilities for interspecific light partitioning to promote biomass accumulation at the stand level. Even if light would be limiting and more diverse stands could fill niches at the lower end of the light availability gradient, these possibly do not contribute much to productivity.

While leaf area index was unresponsive to tree species diversity in our study, leaf litter fall collected in litter traps emptied at monthly intervals increased with tree species richness, in the same plots [52]. Similarly, forest floor leaf litter pools were consistently larger in more species rich plots (S. Trogisch, unpublished). These contrasting responses can be reconciled considering two possibilities: First, specific leaf area may have been lower in more species rich plots. This is likely since there was a trend towards a higher abundance of evergreen species, and because higher biomass and lower light may have forced trees to adopt a more conservative, competitive growth strategy. Second, leaf turnover in plots with higher species richness could be accelerated, i.e. leaf area production could be higher without manifesting as higher standing leaf area. It is conceivable that shorter-lived leaves in more diverse plots were also more productive per unit of time, which could lead to a higher C assimilation with the same community LAI as in less diverse plots.

Canopy height and gap fraction are important for rainfall interception and splash erosion. The gap fraction is linked to leaf area, but structural differences in the canopy such as clumping of leaves can alter this relation [53]. In our study, however, no effects of species richness on canopy gap fraction were detected. Interception of rain drops can moderate their kinetic energy, unless the intercepted water is channeled in a way that results in larger drop sizes with higher terminal velocity upon impact at the soil surface. For the plots we investigated, Geissler et al. [20] reported increased throughfall kinetic energy at higher species diversity and attributed this effect in part to an increased canopy height and therefore also increased drop height. This effect is compatible with the absence of effects of species richness on gap fraction as observed in the present study. In fact, there was a trend-wise increase in gap fraction with species richness in the summer assessment (P = 0.14 for richness fitted before age; model-predicted means of 2% and 3% gap fraction at 10 and 20 canopy tree species). Also, Geissler et al. [20] measured LAI by hemispheric photography in a subset of plots, and a re-analysis of their data revealed the same trend we found, i.e. LAI did not change with tree species numbers. We thus are confident that also this pattern is robust.

Overall, our study demonstrates that tree species richness enhanced stand productivity but not community leaf area in a subtropical forest. The divergent responses of leaves and wood may be related to a functional decoupling of C storage and actively photosynthesizing leaf area not found to this extent in herbaceous systems. Alternatively, the same community leaf area might have assimilated more C per time interval in more than in less diverse plots because of differences in leaf turnover and productivity or because of differences in the display of leaves in vertical and horizontal space. Indeed, increased carbon storage in aboveground biomass was associated with a higher variance in tree size, with the latter driven by interspecific rather than by intraspecific differences.

## Supporting Information

S1 Data Stand basal area (2008 and 2012), leaf area index (spring and summer 2012), and tree aboveground carbon stocks in dependence of plot successional age and diversity (species numbers, functional trait diversity, and phylogenetic diversity). (CSV)

S1 Fig ΔSBA as function of tree species richness and stand age. Line represent the linear regression (ignoring stand age) between species richness and ΔSBA (a). Structural equation model (SEM) for ΔSBA (b) in dependence of stand age, tree diversity and tree density. ΔSBA increased with tree species richness and decreased with stand age. Path diagrams indicate effects of tree species richness on the two dependent variables, either directly or indirectly via tree density. The diagrams show standardized path coefficients (red: positive; blue: negative) and associated statistical significances (*** P<0.001; ** P<0.01; *P<0.05; (*) P<0.1). Variable abbreviations: S = species richness, PD = phylogenetic diversity, FD = functional diversity, DIV = diversity (latent variable related to previous three), AGE = stand age, DENS = tree density, ΔSBA = 2008–2012 increase of stand basal area.
(PDF)
