# Supplementary figures and images for: Tree Diversity Enhances Stand Carbon Storage but Not Leaf Area in a Subtropical Forest
Source: PLoS One. 2016 Dec 9;11(12):e0167771. doi: 10.1371/journal.pone.0167771 (PMC5147976; doi:10.1371/journal.pone.0167771)

(a)

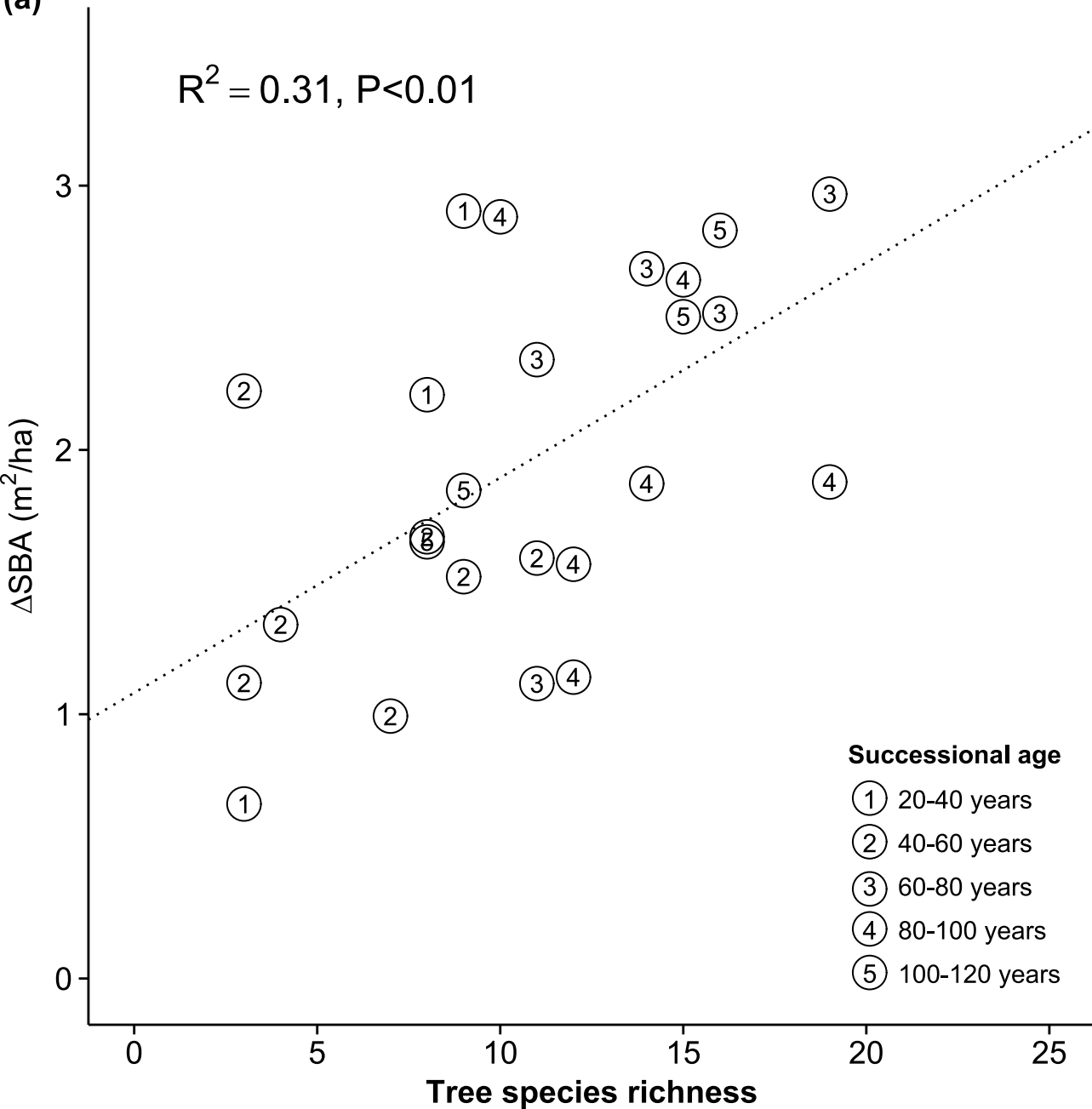

(b)

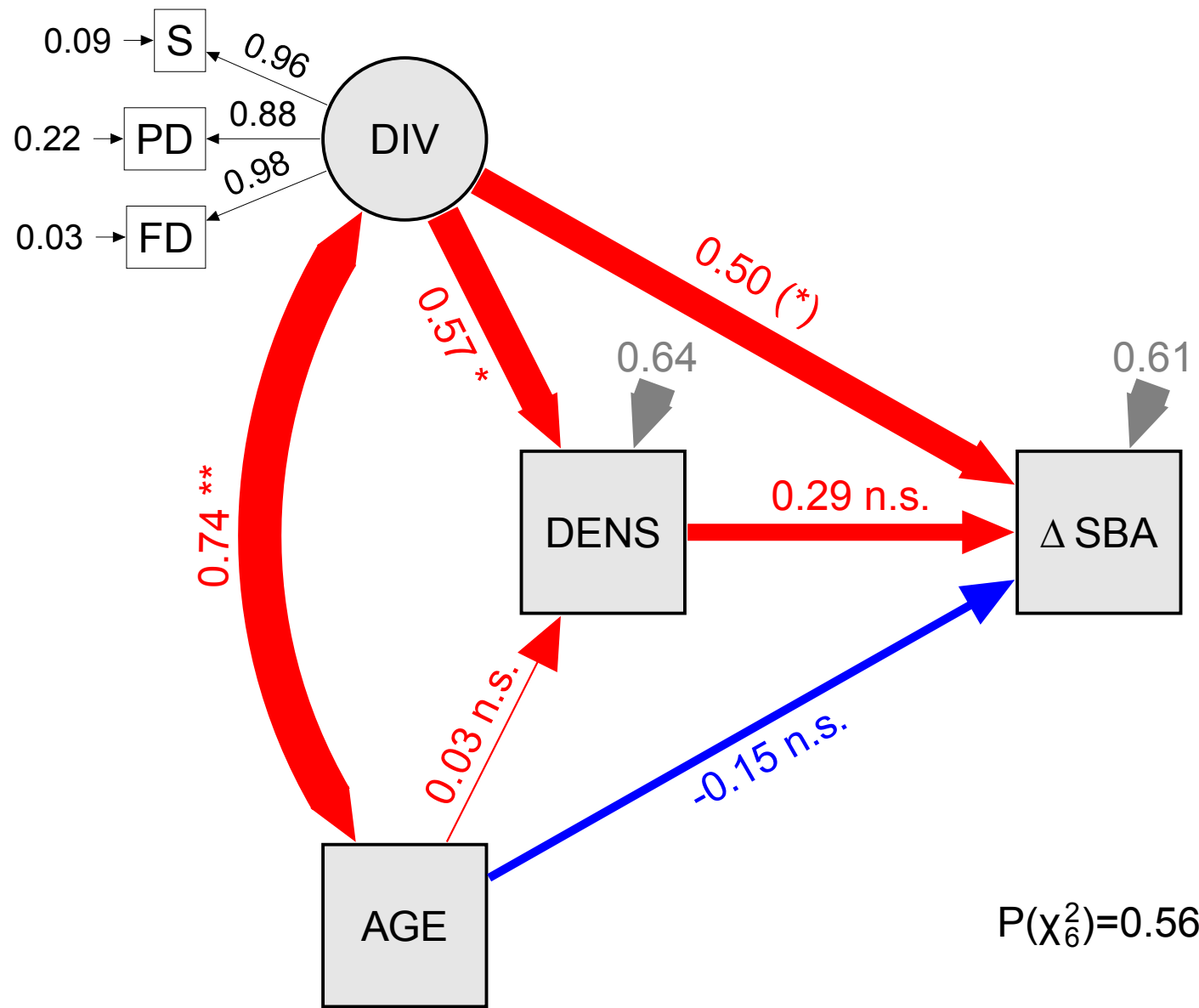

Supplement: S1 Fig — Line represent the linear regression (ignoring stand age) between species richness and ΔSBA (a). Structural equation model (SEM) for ΔSBA (b) in dependence of stand age, tree diversity and tree density. ΔSBA increased with tree species richness and decreased with stand age. Path diagrams indicate effects of tree species richness on the two dependent variables, either directly or indirectly via tree density. The diagrams show standardized path coefficients (red: positive; blue: negative) and associated statistical significances (*** P<0.001; ** P<0.01; *P<0.05; (*) P<0.1). Variable abbreviations: S = species richness, PD = phylogenetic diversity, FD = functional diversity, DIV = diversity (latent variable related to previous three), AGE = stand age, DENS = tree density, ΔSBA = 2008–2012 increase of stand basal area. (PDF) [file pone.0167771.s002.pdf]
